# Progressive distribution adapted neural networks for cross-corpus speech emotion recognition

**DOI:** 10.3389/fnbot.2022.987146

**Published:** 2022-09-15

**Authors:** Yuan Zong, Hailun Lian, Jiacheng Zhang, Ercui Feng, Cheng Lu, Hongli Chang, Chuangao Tang

**Affiliations:** ^1^Key Laboratory of Child Development and Learning Science of Ministry of Education, Southeast University, Nanjing, China; ^2^School of Biological Science and Medical Engineering, Southeast University, Nanjing, China; ^3^School of Cyber Science and Engineering, Southeast University, Nanjing, China; ^4^Affiliated Jiangning Hospital, Nanjing Medical University, Nanjing, China

**Keywords:** cross-corpus speech emotion recognition, speech emotion recognition, deep transfer learning, domain adaptation, deep learning

## Abstract

In this paper, we investigate a challenging but interesting task in the research of speech emotion recognition (SER), i.e., cross-corpus SER. Unlike the conventional SER, the training (source) and testing (target) samples in cross-corpus SER come from different speech corpora, which results in a feature distribution mismatch between them. Hence, the performance of most existing SER methods may sharply decrease. To cope with this problem, we propose a simple yet effective deep transfer learning method called progressive distribution adapted neural networks (PDAN). PDAN employs convolutional neural networks (CNN) as the backbone and the speech spectrum as the inputs to achieve an end-to-end learning framework. More importantly, its basic idea for solving cross-corpus SER is very straightforward, i.e., enhancing the backbone's corpus invariant feature learning ability by incorporating a progressive distribution adapted regularization term into the original loss function to guide the network training. To evaluate the proposed PDAN, extensive cross-corpus SER experiments on speech emotion corpora including EmoDB, eNTERFACE, and CASIA are conducted. Experimental results showed that the proposed PDAN outperforms most well-performing deep and subspace transfer learning methods in dealing with the cross-corpus SER tasks.

## 1. Introduction

Speech is one major way human beings communicate in daily life, which carries abundant emotional information. Consider that if computers were able to understand the emotional states of human beings' speech signals, human-computer interaction would undoubtedly be more natural. Consequently, the research of automatically recognizing emotional states from speech signals, a. k. a., speech emotion recognition (SER) has attracted wide attention among the affective computing, human-computer interaction, and speech signal processing communities (El Ayadi et al., 2011; Schuller, 2018). Over the past several decades, many well-performing SER methods have been proposed and achieved promising performance on widely-used publicly available speech emotion corpora (Zong et al., 2016; Zhang et al., 2017, 2022; Kwon, 2021; Lu et al., 2022). However, it is noted that most of them did not consider the realistic scenario where the training and testing speech signals are possibly recorded by different microphones or in different environments. In this case, a feature distribution mismatch may exist between the training and testing speech samples, and hence the performance of these originally well-performing SER methods may decrease sharply. This brings us a meaningful and more challenging task in SER, i.e., cross-corpus SER. Unlike the conventional SER, the labeled training and unlabeled testing samples in cross-corpus SER come from different speech corpora. Following the naming conventions in cross-corpus SER, we will refer to the training and testing samples/corpora/feature sets as the source and target ones throughout this paper in what follows.

In recent years, researchers have been devoted to the research of cross-corpus SER and proposed many promising methods. Schuller et al. (2010b) may be the first to have investigated this problem, and designed three different normalization methods including speaker normalization (SN), corpus normalization (CN), and speaker-corpus normalization (SCN) to alleviate the feature distribution mismatch between the source and target speech samples. Since that, lots of transfer learning and domain adaptation methods have been successively designed to deal with cross-corpus SER tasks. For example, Hassan et al. (2013) proposed to compensate for the corpus shift by reweighting the source speech samples to deal with cross-corpus SER tasks. A new version of the modified support vector machine (SVM) called importance-weighted SVM (IW-SVM) was designed by incorporating three typical transfer learning methods including kernel mean matching (KMM) (Gretton et al., 2009), unconstrained least-squares importance fitting (uLSIF) (Kanamori et al., 2009), and Kullback-Leibler importance estimation procedure (KLIEP) (Tsuboi et al., 2009) to learn the source sample weights. In the work of Song et al. (2016), Song et al. presented a transfer non-negative matrix factorization (TNMF) for the cross-corpus SER problem. The basic idea of TNMF is to decompose the source and target speech feature sets into different non-negative feature matrices under the guidance of maximum mean discrepancy (MMD) (Borgwardt et al., 2006) and hence the gap between the source and target speech signals described by the non-negative matrices can be alleviated (Liu et al., 2018). Moreover, Liu et al. proposed a domain-adaptive subspace learning (DoSL) model to handle the cross-corpus SER problem. This method measures the distribution gap between the source and the target speech samples through a one-order moment, i.e., the mean value of speech feature vectors. Then a subspace learning model enhanced by the one-order moment regularization term is built to learn a projection matrix to transform the source and target speech sample from the original feature space to the labeled one. The transformed source and target speech samples in such label space would share similar feature distributions. More recently, Zhang et al. (2021) further proposed an extended version of DoSL called joint distribution adaptive regression (JDAR) to align the source and target speech feature distributions to remove their mismatch by considering the marginal distribution gap together with the emotion class aware conditional one. By jointly minimizing both feature distribution gaps, the JDAR model can achieve a better performance than DoSL in dealing with the cross-corpus SER tasks.

On the other hand, deep transfer learning techniques have also been used to cope with the cross-corpus SER tasks. Unlike the transfer subspace learning methods, most deep transfer learning ones try to learn a robust deep neural network to learn corpus invariant features to describe the speech signals. For example, Deng et al. (2014, 2017) proposed a series of unsupervised domain adaptation methods based on autoencoder (AE) to bridge the gap between the source and target speech emotion corpora. The basic idea of these methods is to learn a common subspace through AE instead of widely used subspace learning such that the source and target speech signals have the same or similar feature distributions in the learned subspace. Different from the work of Deng et al. (2014, 2017), Abdelwahab and Busso (2018) proposed to use another deep neural network, i.e., deep belief network (DBN), to investigate the cross-language and cross-corpus SER problem on five speech emotion corpora and the experimental results demonstrated more promising performance than sparse AE and SVM based baseline systems. Recently, adversarial learning-based methods have also been applied to coping with cross-corpus SER tasks. Abdelwahab and Busso (2018) made use of adversarial multi-task training to learn a common representation for training and testing speech feature sets. Two tasks were designed to enable the networks to be robust to the corpus variance. Specifically, one task is to build the relationship between the emotion classes and acoustic descriptors of speech signals. The other is to learn the common representation by enforcing the source and target speech features cannot be distinguished. More recently, Gideon et al. (2019) presented an adversarial discriminative domain generalization (ADDoG) model with the help of domain generalization. Unlike most deep transfer learning methods, the ADDoG model used the speech spectrums as the inputs instead of the handcrafted speech features and simultaneously improved its corpus robustness in multiple speech corpora. Following the work of Gideon et al. (2019), Zhao et al. (2022) also used the speech spectrums as the inputs of the networks to achieve the end-to-end learning manner for cross-corpus SER tasks and proposed a deep transductive transfer regression neural network (DTTRN) with an emotion knowledge guided MMD loss to remove the feature distribution mismatch between the source and target speech corpora.

Inspired by the success of the above deep transfer learning methods, in this paper we also focus on the research of designing deep transfer learning methods to deal with the cross-corpus SER tasks. We propose a novel method called progressive distribution adapted neural networks (PDAN). The basic idea of PDAN is very straightforward, i.e., enabling the deep neural networks to directly learn an emotion discriminative and corpus invariant representations for both source and target original speech signals by leveraging the powerful nonlinear mapping ability and hierarchical structure of deep neural networks. Specifically, we first make use of convolutional neural networks to build the relationship between the source emotion label information and speech spectrums to endow the emotion discriminant ability to PDAN. Then, three feature distribution adapted regularization terms are imposed on different fully connected layers to respectively guide the network to learn the corpus invariant common representations for both speech corpora. To evaluate the effectiveness of the PDAN, we conduct extensive cross-corpus SER experiments on three widely-used speech emotion corpora, i.e., EmoDB (Burkhardt et al., 2005), eNTERFACE (Martin et al., 2006), and CASIA (Zhang and Jia, 2008). Experimental results demonstrate the effectiveness and superior performance of PDAN over recent state-of-the-art transfer learning methods in dealing with cross-corpus SER tasks. In summary, the main contributions of this paper include three folds:

1. We proposed a novel end-to-end deep transfer learning model called PDAN to cope with cross-corpus SER tasks. Unlike most existing methods, PDAN can directly learn the corpus invariant and emotion discriminative speech features from the original speech spectrums by resorting to the nonlinear mapping ability of deep neural networks.
2. We presented a new idea of progressively adapting the feature distributions between the source and target speech samples for the proposed PDAN by designing three different derived MMD loss functions.
3. Extensive cross-corpus SER tasks are designed to evaluate the proposed PDAN method. By deeply analyzing the experimental results, several interesting findings and discussions are given in our paper.

## 2. Proposed method

### 2.1. Overall picture and notations

**Figure 1:**
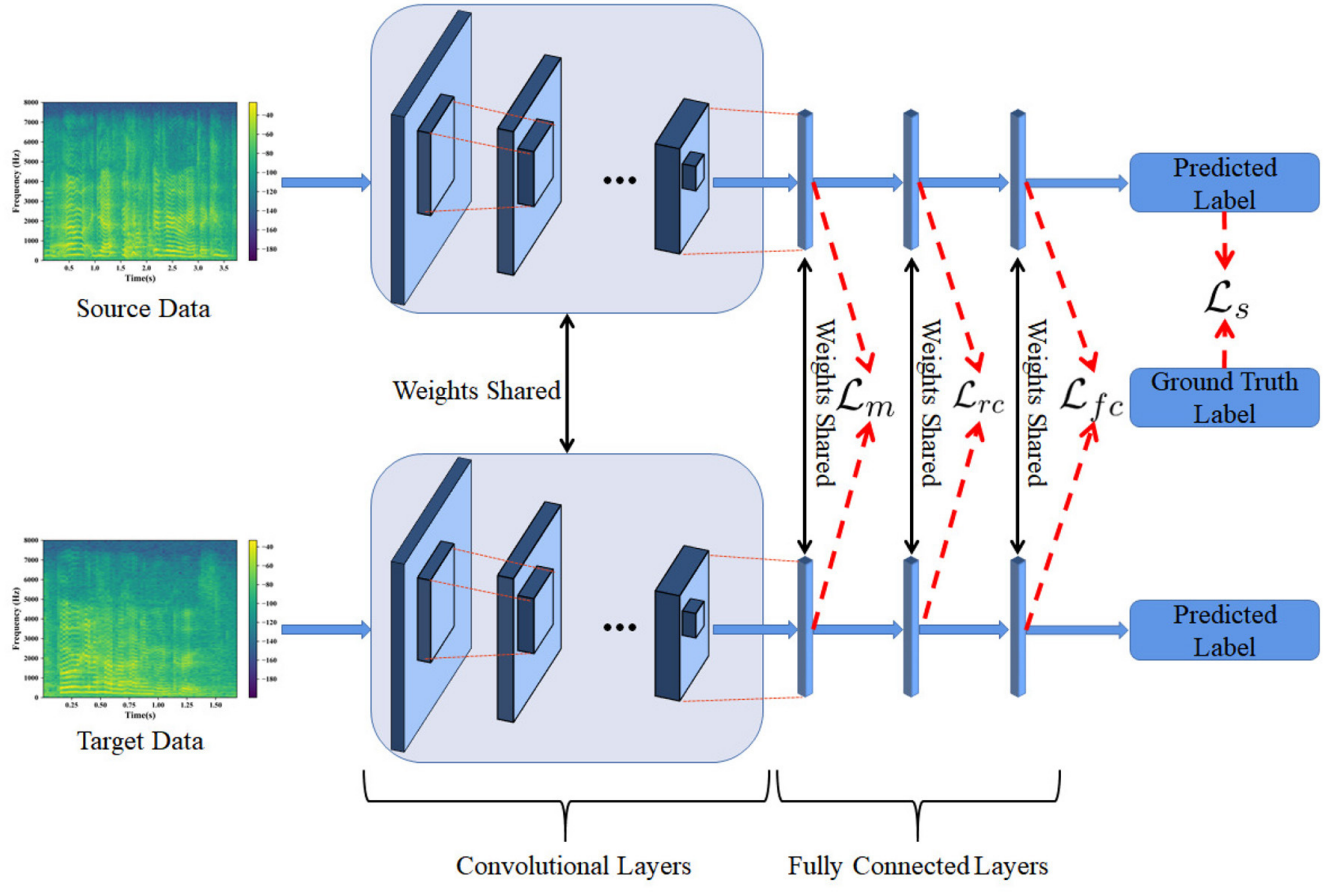
The overview of progressive distribution adapted neural networks (PDAN). The PDAN uses the speech spectrums as the inputs and directly builds the relationship between the emotion labels and speech signals. It consists of several convolutional layers and three fully connected (FC) layers and is trained under the guidance of the combination of four loss functions, i.e., emotion discriminative loss $L_{s}$, marginal distribution adapted loss $L_{m}$, rough emotion class aware conditional distribution adapted loss $L_{rc}$, and fine emotion class aware conditional distribution adapted loss $L_{fc}$.

In this section, we address the proposed PDAN model in detail and also show how to use PDAN to deal with cross-corpus SER tasks. To this end, we draw a picture shown in Figure 1 to illustrate the basic idea and overall structure of the proposed PDAN. To make the readers better understand this paper, we first introduce some necessary notations which are used in Figure 1 for formulating PDAN. The speech spectrums of source and target speech samples are denoted by $D_{s}=\left\{X_{1}^{s},\cdots \;,X_{N_{s}}^{s}\right\}$ and $D_{t}=\{X_{1}^{t},\cdots ,X_{N_{t}}^{t}\}$, respectively, where *N*_*s*_ and *N*_*t*_ are the source and target sample numbers. According to the task setting of cross-corpus SER, the source emotion labels are given, while the target ones are entirely unknown. Hence, we denote the source emotion labels by $Y^{s}=\left\{{\text{y}}_{1}^{s},\cdots \;,{\text{y}}_{N_{s}}^{s}\right\}$. Note that the *i*^*th*^ sample's emotion label ${\text{y}}_{i}^{s}\in {\mathbb{R}}^{C\times 1}$ is a one-hot vector whose *k*^*th*^ entry would be 1 while the others are all 0 if its corresponding label was *k*^*th*^ of *C* emotions.

### 2.2. Formulating PDAN

As described in Sect. Introduction, the basic idea of PDAN is very straightforward, i.e., building an **emotion discriminative** and **corpus invariant** end-to-end neural network for cross-corpus SER. To achieve this goal, we first construct a convolutional neural network (CNN) consisting of a set of convolutional layers and three fully connected (FC) layers to serve as the basic structure of PDAN. Then, to achieve the goal of end-to-end learning, we transform the original speech signals into spectrums to serve as the inputs of the PDAN. Note that in PDAN, the source and target speech spectrums will be simultaneously fed to train the PDAN, which can also be interpreted as inputting them into two weight-shared CNNs shown in Figure 1. Subsequently, it is clear to see from Figure 1 that our PDAN has four major loss functions to guide the network training, i.e., $L_{s}$, $L_{m}$, $L_{rc}$, and $L_{fc}$, respectively, which correspond to the basic idea of the proposed PDAN. The first loss function is called emotion discriminative loss denoted by $L_{s}$, which is designed for enabling the network to be **emotion discriminative** and can be formulated as


(1) $${ℒ}_{s}=\frac{1}{N_{s}}\sum_{i=1}^{N_{s}}J_{CE}(g_{3}(g_{2}(g_{1}(f(X_{i}^{s})))),y_{i}^{s}),$$

where $J_{CE}$ is the cross-entropy loss bridging the source speech spectrums and their corresponding emotion labels, *g*_1_, *g*_2_, and *g*_3_ are the parameters of fully connected layers, and *f* denotes the parameters of the convolutional layers, respectively.

As for the resting loss functions, they aim to improve the robustness of the speech features learned by PDAN to the **corpus invariance**. To this end, based on the MMD criterion (Borgwardt et al., 2006), we first design marginal distribution adapted loss $L_{m}$ and impose it on the first FC layer in PDAN, which is formulated as follows:


(2) $${ℒ}_{m}=\|\frac{1}{N_{s}}\sum_{i=1}^{N_{s}}\Phi (g_{1}(f(X_{i}^{s})))-\frac{1}{N_{t}}\sum_{i=1}^{N_{t}}\Phi (g_{1}(f(X_{i}^{t}))){\|}_{ℋ}^{2},$$

where $L_{m}$ is the square of the original MMD function and can be used to measure the marginal distribution difference between the source and target feature sets, Φ(·) is the kernel mapping operator, and $∥\cdot {∥}_{H}$ means the inner product in such reproduced kernel Hilbert space (RKHS).

Secondly, we design a fine emotion class aware conditional distribution adapted loss $L_{fc}$, which is added to regularize the last FC layer and can be expressed as follows:


(3) $$\begin{array}{l} {ℒ}_{fc}=\frac{1}{C}\sum_{j=1}^{C}\|\frac{1}{N_{s_{j}}}\sum_{i=1}^{N_{s_{j}}}\Phi (g_{3}(g_{2}(g_{1}(f(X_{i}^{s})))))\\ \;-\frac{1}{N_{t}}\sum_{i=1}^{N_{t_{j}}}\Phi (g_{3}(g_{2}(g_{1}(f(X_{i}^{t}))))){\|}_{ℋ}^{2}, \end{array}$$

where $X_{i}^{s_{j}}$ and $X_{i}^{t_{j}}$ correspond to the speech samples belonging to the *j*^*th*^ emotion and *N*_*s*_*j*__ and *N*_*t*_*j*__ denote their sample numbers satisfying *N*_*s*_1__+⋯+*N*_*s*_*C*__ = *N*_*s*_ and *N*_*t*_1__+⋯+*N*_*t*_*C*__ = *N*_*t*_, respectively. Hence, it is clear that $L_{fc}$ can be used to measure the fine emotion class aware conditional feature distribution gap between the source and target speech features.

Finally, we consider designing a rough emotion class aware conditional distribution adapted regularization term, i.e., $L_{rc}$, to guide the feature learning in the second FC layer, whose formulation is as follows:


(4) $$\begin{array}{l} {ℒ}_{rc}=\frac{1}{C_{r}}\sum_{j=1}^{C_{r}}\|\frac{1}{N_{s_{j}}}\sum_{i=1}^{N_{s_{j}}}\Phi (g_{2}(g_{1}(f(X_{i}^{s}))))\\ \;-\frac{1}{N_{t}}\sum_{i=1}^{N_{t_{j}}}\Phi (g_{2}(g_{1}(f(X_{i}^{t})))){\|}_{ℋ}^{2}, \end{array}$$

where *C*_*r*_ < *C* can be called a rough emotion class number.

**Figure 2:**
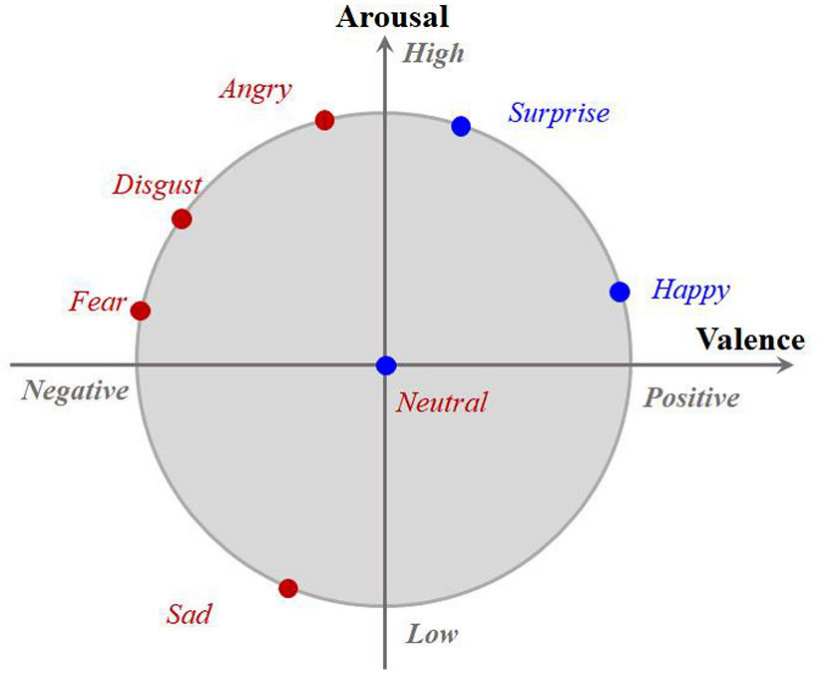
The 2D arousal-valence emotion wheel proposed by Yang et al. (2022). It consists of two dimensions, where the horizontal axis denotes the degree of valence while the vertical axis corresponds to the arousal. Each typical discrete emotion can be mapped to one point in the emotion wheel according to its corresponding valence and arousal values.

Note that $L_{rc}$ shown in Equation (4) looks like a new measurement of conditional distribution mismatch between the source and target speech features, which is so similar to $L_{fc}$ in Equation (3). However, they are actually very different. Specifically, in $L_{rc}$, a set of emotion classes involved in cross-corpus SER will merge together and then the conditional MMD is calculated. This is motivated by the work of the valance-arousal emotion wheel proposed by Yang et al. (2022) shown in Figure 2. As Figure 2 shows, it is clear to see that most of the existing typical emotions are all high-arousal and only a few emotions, e.g., *Sad*, are low-arousal. It is also interesting to see that along the valence dimension, the separability among these emotions would be significantly improved. For example, we can observe from Figure 2 that *Angry*, *Disgust*, and *Fear* are low-valence, while *Surprise* and *Happy* are high-valence although they all belong to the high-arousal ones. Inspired by the above observations, we propose to align the rough emotion-aware conditional distributions with respect to the valence dimension in the second FC layer and hence design $L_{rc}$ to further improve the corpus invariance of the proposed PDAN together with the resting two ones. It should be noticed that since the features in shallow layers have limited discriminative ability, it may be a tough task to directly align the fine emotion class aware conditional distribution gap between the source and target speech features together with the marginal one in the first FC layer. Therefore, we assign the fine emotion class aware conditional distribution term to the last FC layer instead of the first one because such features in the deepest FC layer would be more emotion-discriminative. According to the granularity of the emotion class information used in calculating these three feature distribution adapted terms, it can be seen that the feature distribution adaption operations of PDAN are present in a progressive way. This is why we call the proposed method PDAN.

Under the above considerations, we are able to arrive at the optimization problem of the proposed PDAN by jointly minimizing the four well-designed losses, which can be expressed as follows:


(5) $$\underset{f,g_{1},g_{2},g_{3}}{\text{min}}{ℒ}_{total}={ℒ}_{s}+\lambda_{1}{ℒ}_{m}+\lambda_{2}{ℒ}_{rc}+\lambda_{3}{ℒ}_{fc},$$

where λ_1_, λ_2_, and λ_3_ are the trade-off parameters controlling the balance among the four losses.

### 2.3. Optimization of PDAN

Since the calculation of two conditional distribution adapted loss needs the target label information, we optimize the optimization problem of PDAN by using an alternated direction method. Specifically, we first randomly initialize the parameters of PDAN, i.e., *f*, *g*_1_, *g*_2_, and *g*_3_, and then predict the pseudo emotion labels of target speech samples denoted by ${\text{L}}_{t}^{p}$. Subsequently, perform the following two major steps until convergence:

1. According to ${\text{L}}_{t}^{p}$, calculate the loss functions $L_{total}$ and update the parameters of PDAN, i.e., *f*, *g*_1_, *g*_2_, and *g*_3_, by the typical optimization algorithm, e.g., SGD and Adam.
2. Fix *f*, *g*_1_, *g*_2_, and *g*_3_, and update the pseudo target emotion labels ${\text{L}}_{t}^{p}$.

Note that in PDAN, the kernel trick can be used to effectively calculate three MMD based losses, which can be formulated as follows:


(6) $$\begin{array}{l} \text{MM}{\text{D}}^{2}({\text{X}}^{s},{\text{X}}^{t})=\;\|\frac{1}{N_{s}}\sum_{i=1}^{N_{s}}\Phi ({\text{x}}_{i}^{s})))-\frac{1}{N_{t}}\sum_{i=1}^{N_{t}}\Phi ({\text{x}}_{i}^{t}))){\|}_{ℋ}^{2},\\ \;=\frac{N_{s}}{N_{s}(N_{s}-1)}\sum_{i\neq j}^{N_{s}}k({\text{x}}_{i}^{s},{\text{x}}_{j}^{s})+\frac{1}{N_{t}(N_{t}-1)}\\ \;\sum_{i\neq j}^{N_{t}}k({\text{x}}_{i}^{t},{\text{x}}_{j}^{t})-\frac{2}{N_{s}N_{t}}\sum_{i,j=1}^{N_{s},N_{t}}k({\text{x}}_{i}^{s},{\text{x}}_{j}^{t}), \end{array}$$

where *k*(·) is a kernel function replacing the inner product operation between vectors in RKHS produced by Φ(·) with calculating a predefined function, and ${\text{x}}_{i}^{s}$ and ${\text{x}}_{i}^{t}$ are the *i*^*th*^ column in **X**^*s*^ and ${\text{X}}_{i}^{t}$.

Finally, we summarize the detailed procedures for updating PDAN in Algorithm 1 such that the readers can better understand how to optimize the proposed PDAN.

**Algorithm 1:** The detailed procedures for updating optimization problem of PDAN in Equation (5).

**Input:** Source Speech Spectrums: $D_{s}=\left\{X_{1}^{s},\cdots \;,X_{N_{s}}^{s}\right\}$,
Target Speech Spectrums: $D_{t}=\left\{X_{1}^{t},\cdots \;,X_{N_{t}}^{s}\right\}$,
Learning Rate: α,
Trade-off Parameters: **λ_1_**, λ_2_, **and** **λ_3_**,
Maximal Iterations: *N*_*max*_.
**Output:** Optimal Network Parameters: $f=\hat{f}$, *g*_1_ = ĝ_1_, *g*_2_ = ĝ_2_, and *g*_3_ = ĝ_3_.
1: Initialize the network parameters: $\tilde{f}$, ${\tilde{g}}_{1}$, ${\tilde{g}}_{2}$, and ${\tilde{g}}_{3}$, and iteration indicator: *iter* = 0.
2: **while** $L_{total}\neq 0\;||\;iter<N_{max}$ **do**
3: *iter* = *iter*+1;
4: Fix *f*, *g*_1_, *g*_2_, and *g*_3_, predict the pseudo label ${\text{L}}_{t}^{p}$;
5: Fix ${\text{L}}_{t}^{p}$, calculate $L_{total}$;
6: Update *f*, *g*_1_, *g*_2_, and *g*_3_:
7: $\nabla_{\theta }\leftarrow \frac{\partial \left(L_{s}+\lambda_{1}L_{m}+\lambda_{2}L_{rc}+\lambda_{3}L_{fc}\right)}{\partial \theta }$, where **θ** = {*f, g*_1_, *g*_2_, *g*_3_};
8: $\theta^{n+1}\leftarrow \theta^{n}-\alpha \nabla_{\theta }$;
9: **end while**

## 3. Experiments

### 3.1. Speech emotion corpora and protocol

In this section, we design extensive cross-corpus SER tasks to evaluate the proposed PDAN method. Three public available speech emotion corpora including EmoDB (Burkhardt et al., 2005), eNTERFACE (Martin et al., 2006), and CASIA (Zhang and Jia, 2008), are chosen. EmoDB is one of the most widely-used German acted speech emotion corpora collected by Burkhardt et al. from TU Berlin, Germany. Ten participants consisting of five women and five men were recruited to simulate seven types of emotions, i.e., *Neutral*, *Angry*, *Fear*, *Happy*, *Sad*, *Disgust*, and *Boredom*, respectively. The total sample number reaches 545 and can be downloaded from the http://www.expressive-speech.net/emodb/. eNTERFACE is an induced audio-video bi-modal emotion database. We only adopted its audio part and the language is English. It consists of 1,257 speech samples from 41 independent speakers comprising six basic emotions, i.e., *Disgust*, *Sad*, *Angry*, *Happy*, *Fear*, and *Surprise*, respectively. CASIA is a Chinese acted speech corpus designed by the Institute of Automation, Chinese Academy of Science. It recruited four speakers including two women and two men to record 1,200 speech samples from six typical emotions, i.e., *Neutral*, *Surprise*, *Angry*, *Happy*, *Fear*, and *Sad*.

By alternatively using either two of these three speech emotion corpora to serve as the source and target domains, six cross-corpus SER tasks are designed denoted by *B* → *E*, *B* → *E*, *B* → *E*, *B* → *E*, *B* → *E*, and *B* → *E*, respectively. Note that *B*, *E*, and *C* are the abbreviations of EmoDB, eNTERFACE, and CASIA. The left and right corpora of the arrow denote the source and target ones in such a cross-corpus SER task. Since these three corpora have inconsistent emotion labeling information, in each task we select the speech samples sharing the same emotion label from the corresponding source and target corpora. To make the readers better know the detail of the sample information in each cross-corpus SER task, we summarize the sample statistics of speech corpora used in all six tasks in Table 1. As for the performance metric, we choose unweighted average recall (UAR) (Schuller et al., 2010b) defined as the accuracy per class averaged by the total emotion class number, which is widely used in evaluating SER methods. For comparison purpose, five typical transfer subspace learning methods, i.e., Transfer Component Analysis (TCA) (Pan et al., 2010), Geodesic Flow Kernel (GFK) (Gong et al., 2012), Subspace Alignment (SA) (Fernando et al., 2013), Domain Adaptive Subspace Learning (DoSL) (Liu et al., 2018), and Joint Distribution Adaptive Regression (JDAR) (Zhang et al., 2021), respectively, and four deep transfer learning methods, i.e., Deep Adaptation Networks (DAN) (Long et al., 2015), Domain-Adversarial Neutral Network (DANN) (Ajakan et al., 2014), Deep-CORAL (Sun and Saenko, 2016), and Deep Subdomain Adaptation Network (DSAN) (Zhu et al., 2020), respectively, are included.

**Table 1:** The sample statistics of EmoDB (B), eNTERFACE (E), and CASIA (C) corpora used in the designed six cross-corpus SER tasks.

**Tasks**	**Speech corpus (# Samples belonging to each emotion)**	**Total**
*B* → *E*	B (Angry: 127, Sad: 62, Fear: 69, Happy: 71, Disgust: 46)	375
*E* → *B*	E (Angry: 211, Sad: 211, Fear: 211, Happy: 208, Disgust: 211)	1,052
*B* → *C*	B (Angry: 127, Sad: 62, Fear: 69, Happy: 71, Neutral: 79)	408
*C* → *B*	C (Angry: 200, Sad: 200, Fear: 200, Happy: 200, Neutral: 200)	1,000
*E* → *C*	E (Angry: 211, Sad: 211, Fear: 211, Happy: 208, Surprise: 211)	1,052
*C* → *E*	C (Angry: 200, Sad: 200, Fear: 200, Happy: 200, Surprise: 200)	1,000

### 3.2. Implementation details

First, as for the subspace learning comparison methods, we choose two types of speech feature sets, i.e., IS09 (Schuller et al., 2009) and IS10 (Schuller et al., 2010a) to describe speech signals, respectively. The IS09 feature set consists of 384 elements including 16 ×2 acoustic low-level descriptors (LLDs) such as fundamental frequency (F0), zero-crossing rate (ZCR), and Mel-frequency cepstrum coefficient (MFCC), and their first order difference, and their 12 corresponding functions such as maximal value, mean value, and minimal value. The IS10 feature set has 1,582 elements which are obtained by applying 21 statistical functions to 38 LLDs and their first order derivatives plus 2 single features about F0 (the number of onsets and tern duration) and discarding 16 zero-information features (e.g., minimum F0). The detailed information of these two feature sets are referred to in the works of Schuller et al. (2009) and Schuller et al. (2010a), respectively. In the experiments, the openSIMLE toolkit (Eyben et al., 2010) is used to extract the IS09 and IS10 feature sets. The hyper-parameters of all the subspace learning methods are set as follows:

1. **TCA**, **GFK**, and **SA**: A hyper-parameter, i.e., the reduced dimension denoted by *d*, needs to be set for TCA, GFK, and SA. In the experiments, we search the *d* from a parameter interval [5:5:*d*_*max*_], where *d*_*max*_ is the maximal dimension reduced by these three methods in each experiment.
2. **DoSL** and **JDAR**: There are two hyper-parameters in DoSL and JDAR methods, i.e., λ and μ. They are used to control the balance between the original regression loss function and two regularization terms including feature selection and feature distribution difference alleviation terms. In the experiments, they are both searched from the parameter interval [5:5:100]. In addition, since the JDAR method needs to iteratively predict the pseudo emotion labels of the target speech signals and calculate the emotion class aware conditional distribution gap between the source and target speech feature sets, we set the iterations as 5 for JDAR in all the cross-corpus SER tasks.

Second, as for the deep learning methods including our PDAN, we first transform the original speech signals into speech spectrums to serve as the inputs of all the methods. Specifically, for each speech sample from the emotion corpora, we set the frame size and overlap as 350 and 175 sampling points, respectively, and then all the speech frames windowed by the Hamming function were transformed to spectrums by using Fourier transformation to compose the speech spectrums. Note that in speech spectrum generation, the sampling frequencies used for EmoDB, eNTERFACE, and CASIA are 16, 44, and 16 kHz, respectively. In the implementations of all the deep learning methods, the Adam optimizer is used to train the model. Its three parameters, i.e., β_1_, β_2_, and weight decay λ are set as 0.9, 0.999, and 0.005, respectively. During the training stage, the batch size and the initial learning rate are set to 32 and 0.0002, respectively. AlexNet (Krizhevsky et al., 2012) is served as the CNN backbone of all the deep learning methods and only the neuron number of the last fully connected layer is reset as the one involving emotion class number in each cross-corpus SER task. Moreover, since most of the comparison methods adopt MMD losses, following the work of Long et al. (2015) and Zhu et al. (2020), we use the mixed Gaussian function to serve as the kernel function, i.e., $\text{K}=\overset{5}{\underset{i=1}{\sum }}K_{i}$, where $K_{i}\left(\text{u},\text{v};\sigma_{i}\right)=e^{\frac{-∥\text{u}-\text{v}{∥}^{2}}{2\sigma_{i}^{2}}}$, where σ_*i*_ denotes the bandwidth and its value range is [2, 4, 8, 16, 32]. Finally, the trade-off parameter of each comparison methods is set as follows:

1. **DAN** and **DSAN**: There is only one trade-off parameter in DAN and DSAN. We set its interval as [0.001, 0.005, 0.01, 0.05, 0.1, 0.5].
2. **DANN**: DANN also has only one trade-off parameter. We set its searching range as [0.001, 0.003, 0.005, 0.01, 0.05, 0.1, 0.5].
3. **Deep-CORAL**: Similar to the above deep transfer learning methods, one trade-off parameter in Deep-CORAL needs to be set. In the experiments, its interval is [1, 10, 20, 30, 50, 100].
4. **PDAN**: The proposed PDAN has three trade-off parameters, i.e., λ_1_, λ_2_, and λ_3_. We search them from [0.001, 0.005, 0.01, 0.05, 0.1, 0.5] throughout all the tasks. Moreover, since the proposed PDAN needs to update the target labels in the optimization, in the training stage we will fix the network parameters and update the target labels at the end of each epoch. In addition, we set the rough class number *C*_*r*_ = 2 and divide the original emotions into two rough classes including *High*-*Valence* (*Happy*, *Surprise*, and *Neutral*) and *Low*-*Valence* (*Angry*, *Sad*, *Fear*, and *Disgust*).

Finally, since the target label information in cross-corpus SER is entirely unknown, it is not possible to use the validation set to determine the optimal model during the training stage for the transfer learning methods. Therefore, to offer a fair comparison, we follow the tradition of transfer learning method evaluation and report the best results corresponding to the best trade-off parameters for all the methods in the experiments.

### 3.3. Results and discussions

Experimental results are given in Table 2. From Table 2, several interesting observations can be obtained. First, it can be clearly seen that the proposed PDAN method achieved the best average UAR reaching 42.83% among all the transfer learning methods, which has an increase of 1.06% compared with the second best well-performing method (JDAR + IS10 feature set). Moreover, among all the six cross-corpus SER tasks, our PDAN performs better than all the comparison methods in three others, i.e., E → B, B → C, C → B, respectively. Although the proposed PDAN did not achieve the best performance in the resting three tasks, it can be seen from the comparisons that the results obtained from our method are very competitive against the best-performing comparison methods, e.g., 36.19% (PDAN) v.s. 37.95% (JDAR + IS10 feature set) in task B → E. These observations demonstrated the superiority of the PDAN over recent state-of-the-art transfer subspace learning and deep transfer learning methods in dealing with cross-corpus SER tasks.

**Table 2:** The experimental results of all the transfer learning methods for six cross-corpus SER tasks, in which the best results are highlighted in bold.

**Method**		**B → E**	**E → B**	**B → C**	**C → B**	**E → C**	**C → E**	**Average**
Subspace Learning (IS09 Feature Set)	SVM	28.93	23.58	29.60	35.01	26.10	25.14	28.06
	TCA	30.52	44.03	33.40	45.07	31.10	32.32	36.07
	GFK	32.11	42.48	33.10	48.08	32.80	28.13	36.17
	SA	33.50	43.89	35.80	49.03	32.60	28.17	36.33
	DoSL	36.12	38.95	34.40	45.75	30.40	31.59	36.20
	JDAR	36.33	39.97	31.10	46.29	32.40	31.50	36.27
Subspace Learning (IS10 Feature Set)	SVM	34.50	28.13	35.30	35.29	24.30	26.81	30.73
	TCA	32.60	44.53	40.50	51.47	33.20	29.77	38.68
	GFK	36.01	40.11	40.00	45.93	33.00	29.09	37.35
	SA	35.65	43.92	37.50	47.06	32.10	30.61	37.80
	DoSL	36.82	43.33	36.80	48.45	**35.60**	33.91	39.15
	JDAR	**37.95**	47.80	42.70	48.97	**35.60**	**37.58**	41.76
Deep Learning	AlexNet	29.49	31.03	32.90	42.23	27.59	26.30	31.59
	DAN	36.13	40.41	39.00	49.85	29.00	31.47	37.64
	DANN	33.38	43.68	39.20	53.71	29.80	29.25	38.05
	Deep-CORAL	35.03	43.38	38.30	48.28	31.00	30.89	37.81
	DSAN	36.19	46.90	40.30	50.69	29.70	32.61	39.41

Second, by comparing the results obtained by the subspace learning methods with IS09 and IS10 feature sets, it can be found that most methods would achieve better performance when using the IS10 feature set to describe speech signals. For example, JDAR achieved the average UAR of 41.76% when using the IS10 feature set, while its average UAR would decrease to 36.27% if the feature set used to describe speech instead adopted IS09. This may attribute to the limited representation ability of the IS09 feature set compared to IS10. According to the works of Schuller et al. (2009, 2010a), it can be known that the IS10 feature set contains more acoustic LLDs (38) and introduces more statistical functions (21) than IS09 (32 and 12), which leads to a greater capacity of IS10 in describing speech signals. Hence, the transfer subspace learning methods may learn more discriminative representations from the IS10 feature set in coping with cross-corpus SER tasks.

Third, it is also interesting to see that several transfer subspace learning methods using the IS10 feature set, e.g., DoSL and JDAR, outperformed most deep transfer learning ones. This may attribute to the more powerful discriminative ability of the IS10 feature set compared with the features directly learned from the speech spectrums by the deep neural networks. Note that besides the corpus invariant ability, the discriminative one is also an important factor affecting the performance of transfer learning methods, which can be supported by the comparison between the results of IS09 and IS10 feature sets. Consequently, with IS10 as the feature set, several subspace learning methods may achieve better performance than the deep learning ones in coping with the cross-corpus SER tasks.

Last but not least, by deeply comparing the results of all the methods for tasks C → B and B → C and others, it is interesting to see that most methods usually performed better in these two tasks. This may be caused by the difference of emotion-induced methods among these three speech corpora. Specifically, it can be found from the works of Burkhardt et al. (2005), Martin et al. (2006), and Zhang and Jia (2008) that EmoDB and CASIA are both acted speech corpora, while eNTERFACE is an induced one. In other words, the emotional speech samples of EmoDB and CASIA are both acted by the speakers, which are quite different from the ones in eNTEFACE. In eNTERFACE, several stimulus materials were first used to induce the speakers' natural emotions, and then their speech signals were synchronously recorded.

### 3.4. Ablation study

As Figure 1 and Equation (5) show, the proposed PDAN have a set of progressive distribution adapted regularization terms, which enable the network to learn the corpus invariant features for cross-corpus SER and are different from other deep transfer learning methods, e.g., DAN, DANN, and DSAN. Specifically, the proposed progressive distribution adapted regularization term designed for our PDAN has two major advantages. First, besides widely-used marginal and fine class aware conditional distribution adaptions, we also introduce a rough emotion class aware conditional one to benefit the alleviation of feature distribution difference between the source and target speech emotion corpora. Second, these distribution adapted terms are added to regularize different FC layers of CNN to guide the corpus invariant feature learning, which takes full advantage of the hierarchical structure of deep neural networks. It is clear to see that the computation of marginal distribution adapted term does not need the emotion label information, while the two conditional ones are opposite. Moreover, the fine class aware conditional one needs more precise emotion label information of the speech samples compared with the rough one. Consequently, following the fact that the features learned in the deeper layers would have more discriminative ability with respect to the depth of neural network, we propose a progressive regularization method to make full use of these three terms, i.e., adding the marginal one to the first FC layer, the rough conditional one to the second FC layer, and the fine conditional one to the last FC layer, respectively.

To see whether the designed progressive adapted regularization terms are indeed effective, we conduct additional experiments by removing one or two of the rough emotion class aware conditional distribution adapted term $L_{rc}$ and fine emotion class aware one $L_{fc}$ to obtain the new total loss function to train the PDAN. The reduced versions of PDAN are denoted by $L_{s}+L_{m}$ and $L_{s}+L_{m}+L_{fc}$, respectively. The experimental results are shown in Table 3. From Table 3, it can be found that the PDAN trained under the guidance of $L_{s}+L_{m}+L_{rc}+L_{fc}$ and $L_{s}+L_{m}+L_{fc}$ performed promisingly better than the one associated with $L_{s}+L_{m}$ in all six cross-corpus SER tasks. This observation indicates that the performance of PDAN introducing the conditional distribution adaptions would be remarkably increased compared with merely using the marginal distribution adaption. Moreover, it can also be seen that the results achieved by PDAN under the guidance of $L_{s}+L_{m}+L_{rc}+L_{fc}$ are better than $L_{s}+L_{m}+L_{fc}$, which demonstrates the effectiveness of further introducing the rough conditional distribution adaption and the superiority of the proposed progressive distribution adaptions used in PDAN for dealing with cross-corpus SER tasks.

**Table 3:** Experimental results of PDAN with different total loss functions for six cross-corpus SER tasks, in which the best results are highlighted in bold.

**Method**	**B → E**	**E → B**	**B → C**	**C → B**	**E → C**	**C → E**	**Average**
$L_{s}+L_{m}$	34.36	43.39	37.50	48.89	30.00	30.12	37.38
$L_{s}+L_{m}+L_{fc}$	35.16	48.96	41.40	54.96	32.70	32.98	41.03
$L_{s}+L_{m}+L_{rc}+L_{fc}$	**36.19**	**53.78**	**42.90**	**56.88**	**33.70**	**33.54**	**42.83**

## 4. Conclusion

In this paper, we have proposed a novel deep transfer learning method called progressive distribution adapted neural networks (PDAN) to deal with the problem of cross-corpus SER. Unlike existing deep transfer learning methods, PDAN absorbs the knowledge of the emotion wheel and makes full use of the hierarchical structure of deep neural networks. Specifically, we design a progressive distribution adapted regularization term consisting of a marginal distribution adaption and two different types of conditional distribution adaptions to layer-by-layer guide the feature learning of PDAN. Hence, PDAN can learn the emotion discriminative and corpus invariant features for speech signals and be effective to deal with cross-corpus SER tasks. Extensive experiments on three widely-used speech emotion corpora were conducted to evaluate the performance of the proposed PDAN. Experimental results showed that the proposed PDAN can achieve a more satisfactory overall performance than recent state-of-the-art transfer subspace learning and deep transfer learning methods in coping with cross-corpus SER tasks.

## Data availability statement

Publicly available datasets were analyzed in this study. This data can be found here: EmoDB, http://emodb.bilderbar.info/start.html, eNTERFACE, http://www.enterface.net/enterface05, and CASIA, http://www.chineseldc.org.

## Author contributions

YZ: conceptualization, methodology, and funding acquisition. YZ and HL: writing and original draft preparation. HL and JZ: formal analysis. EF: investigation. CL: resources and data curation. HC and CT: review and editing. All authors have read and agreed to the published version of the manuscript.

## Funding

This work was supported in part by the Natural National Science Foundation of China (NSFC) under the Grant Nos. U2003207, 61902064, and 62076195, in part by the Jiangsu Frontier Technology Basic Research Project under the Grant No. BK20192004, in part by the Zhishan Young Scholarship of Southeast University, and in part by the Yangtze River Delta Regional Leading Talents Research Project on Immunization under the Grant No. CSJP005.

## Conflict of interest

The authors declare that the research was conducted in the absence of any commercial or financial relationships that could be construed as a potential conflict of interest.

## Publisher's note

All claims expressed in this article are solely those of the authors and do not necessarily represent those of their affiliated organizations, or those of the publisher, the editors and the reviewers. Any product that may be evaluated in this article, or claim that may be made by its manufacturer, is not guaranteed or endorsed by the publisher.

## References

[B1] Abdelwahab M. Busso C. (2018). Domain adversarial for acoustic emotion recognition. IEEE/ACM Trans. Audio Speech Lang. Process. 26, 2423–2435. 10.1109/TASLP.2018.2867099

[B2] Ajakan H. Germain P. Larochelle H. Laviolette F. Marchand M. (2014). Domain-adversarial neural networks. arXiv preprint arXiv:1412.4446. 10.48550/arXiv.1505.07818

[B3] Borgwardt K. M. Gretton A. Rasch M. J. Kriegel H.-P. Schölkopf B. Smola A. J. (2006). Integrating structured biological data by kernel maximum mean discrepancy. Bioinformatics 22, e49-e57. 10.1093/bioinformatics/btl242 16873512

[B4] Burkhardt F. Paeschke A. Rolfes M. Sendlmeier W. F. Weiss B.. (2005). “A database of german emotional speech,” in Proceedings of the 2005 Annual Conference of the International Speech Communication Association (INTERSPEECH) (Lisbon: ISCA), 1517–1520.

[B5] Deng J. Xu X. Zhang Z. Frühholz S. Schuller B. (2017). Universum autoencoder-based domain adaptation for speech emotion recognition. IEEE Signal Process. Lett. 24, 500–504. 10.1109/LSP.2017.2672753

[B6] Deng J. Zhang Z. Eyben F. Schuller B. (2014). Autoencoder-based unsupervised domain adaptation for speech emotion recognition. IEEE Signal Process. Lett. 21, 1068–1072. 10.1109/LSP.2014.2324759

[B7] El Ayadi M. Kamel M. S. Karray F. (2011). Survey on speech emotion recognition: Features, classification schemes, and databases. Pattern Recognit. 44, 572–587. 10.1016/j.patcog.2010.09.020

[B8] Eyben F. Wöllmer M. Schuller B. (2010). “Opensmile: the munich versatile and fast open-source audio feature extractor,” in Proceedings of the 18th ACM International Conference on Multimedia (MM) (Florence: ACM), 1459–1462.

[B9] Fernando B. Habrard A. Sebban M. Tuytelaars T. (2013). “Unsupervised visual domain adaptation using subspace alignment,” in Proceedings of the 2013 IEEE International Conference on Computer Vision (ICCV) (Sydney, NSW: IEEE), 2960–2967.

[B10] Gideon J. McInnis M. G. Provost E. M. (2019). Improving cross-corpus speech emotion recognition with adversarial discriminative domain generalization (ADDoG). IEEE Trans. Affect. Comput. 12, 1055–1068. 10.1109/TAFFC.2019.2916092 35695825 PMC9173710

[B11] Gong B. Shi Y. Sha F. Grauman K. (2012). “Geodesic flow kernel for unsupervised domain adaptation,” in Proceedings of the 2012 IEEE Conference on Computer Vision and Pattern Recognition (CVPR) (Providence, RI: IEEE), 2066–2073.

[B12] Gretton A. Smola A. Huang J. Schmittfull M. Borgwardt K. Schölkopf B. (2009). Covariate shift by kernel mean matching. Dataset Shift Mach. Learn. 3, 5. 10.7551/mitpress/9780262170055.003.0008

[B13] Hassan A. Damper R. Niranjan M. (2013). On acoustic emotion recognition: compensating for covariate shift. IEEE Trans. Audio Speech Lang. Process. 21, 1458–1468. 10.1109/TASL.2013.2255278

[B14] Kanamori T. Hido S. Sugiyama M. (2009). A least-squares approach to direct importance estimation. J. Mach. Learn. Res. 10, 1391–1445. 10.5555/1577069.1755831

[B15] Krizhevsky A. Sutskever I. Hinton G. E. (2012). “Imagenet classification with deep convolutional neural networks,” in Advances in Neural Information Processing Systems (NIPS), Vol. 25 (Lake Tahoe, NV).

[B16] Kwon S. (2021). Mlt-dnet: speech emotion recognition using 1d dilated cnn based on multi-learning trick approach. Expert. Syst. Appl. 167, 114177. 10.1016/j.eswa.2020.114177

[B17] Liu N. Zong Y. Zhang B. Liu L. Chen J. Zhao G.. (2018). “Unsupervised cross-corpus speech emotion recognition using domain-adaptive subspace learning,” in Proceedings of the 2018 IEEE International Conference on Acoustics, Speech and Signal Processing (ICASSP) (Calgary, AB: IEEE), 5144–5148.

[B18] Long M. Cao Y. Wang J. Jordan M. (2015). “Learning transferable features with deep adaptation networks,” in Proceedings of the 2015 International Conference on Machine Learning (ICML) (Lille), 97–105.

[B19] Lu C. Zong Y. Zheng W. Li Y. Tang C. Schuller B. (2022). Domain invariant feature learning for speaker-independent speech emotion recognition. IEEE/ACM Trans. Audio Speech Lang. Process. 30, 2217–2230. 10.1109/TASLP.2022.3178232

[B20] Martin O. Kotsia I. Macq B. Pitas I. (2006). “The enterface'05 audio-visual emotion database,” in Proceedings of the 22nd International Conference on Data Engineering Workshops (Atlanta, GA: IEEE), 8–8.

[B21] Pan S. J. Tsang I. W. Kwok J. T. Yang Q. (2010). Domain adaptation via transfer component analysis. IEEE Trans. Neural Netw. 22, 199–210. 10.1109/TNN.2010.2091281 21095864

[B22] Schuller B. Steidl S. Batliner A. (2009). “The interspeech 2009 emotion challenge,” in Proceedings of the 2009 Annual Conference of the International Speech Communication Association (INTERSPEECH) (Brighton: ISCA).

[B23] Schuller B. Steidl S. Batliner A. Burkhardt F. Devillers L. Müller C.. (2010a). “The interspeech 2010 paralinguistic challenge,” in Proceedings of the 2010 Annual Conference of the International Speech Communication Association (INTERSPEECH) (Makuhari: ISCA), 2794–2797.

[B24] Schuller B. Vlasenko B. Eyben F. Wöllmer M. Stuhlsatz A. Wendemuth A.. (2010b). Cross-corpus acoustic emotion recognition: variances and strategies. IEEE Trans. Affect. Comput. 1, 119–131. 10.1109/T-AFFC.2010.8

[B25] Schuller B. W. (2018). Speech emotion recognition: two decades in a nutshell, benchmarks, and ongoing trends. Commun. ACM. 61, 90–99. 10.1145/3129340

[B26] Song P. Zheng W. Ou S. Zhang X. Jin Y. Liu J.. (2016). Cross-corpus speech emotion recognition based on transfer non-negative matrix factorization. Speech Commun. 83, 34–41. 10.1016/j.specom.2016.07.010

[B27] Sun B. Saenko K. (2016). “Deep coral: correlation alignment for deep domain adaptation,” in Proceedings of the 2016 European Conference on Computer Vision (ECCV) (Amsterdam: Springer), 443–450.

[B28] Tsuboi Y. Kashima H. Hido S. Bickel S. Sugiyama M. (2009). Direct density ratio estimation for large-scale covariate shift adaptation. J. Inf. Process. 17, 138–155. 10.2197/ipsjjip.17.138

[B29] Yang L. Shen Y. Mao Y. Cai L. (2022). “Hybrid curriculum learning for emotion recognition in conversation,” in Proceedings of the Thirty-Sixth AAAI Conference on Artificial Intelligence (AAAI) (AAAI).

[B30] Zhang J. Jiang L. Zong Y. Zheng W. Zhao L. (2021). “Cross-corpus speech emotion recognition using joint distribution adaptive regression,” in Proceedings of the 2021 IEEE International Conference on Acoustics, Speech and Signal Processing (ICASSP) (Toronto, ON: IEEE), 3790–3794.

[B31] Zhang J. T. F. L. M. Jia H. (2008). “Design of speech corpus for mandarin text to speech,” in Proceedings of the Blizzard Challenge 2008 Workshop at INTERSPEECH (Brisbane: ISCA).

[B32] Zhang S. Zhang S. Huang T. Gao W. (2017). Speech emotion recognition using deep convolutional neural network and discriminant temporal pyramid matching. IEEE Trans. Multimedia 20, 1576–1590. 10.1109/TMM.2017.2766843

[B33] Zhang S. Zhao X. Tian Q. (2022). Spontaneous speech emotion recognition using multiscale deep convolutional lstm. IEEE Trans. Affect. Comput. 13, 680–688. 10.1109/TAFFC.2019.2947464

[B34] Zhao Y. Wang J. Ye R. Zong Y. Zheng W. Zhao L. (2022). “Deep transductive transfer regression network for cross-corpus speech emotion recognition,” in Proceedings of the 2022 Annual Conference of the International Speech Communication Association (INTERSPEECH) (Incheon: ISCA).

[B35] Zhu Y. Zhuang F. Wang J. Ke G. Chen J. Bian J.. (2020). Deep subdomain adaptation network for image classification. IEEE Trans. Neural Netw. Learn. Syst. 32, 1713–1722. 10.1109/TNNLS.2020.2988928 32365037

[B36] Zong Y. Zheng W. Cui Z. Li Q. (2016). Double sparse learning model for speech emotion recognition. Electron. Lett. 52, 1410–1412. 10.1049/el.2016.1211

